# SARS-CoV-2 Omicron subvariants progressively adapt to human cells with altered host cell entry

**DOI:** 10.1128/msphere.00338-24

**Published:** 2024-08-27

**Authors:** Yasuteru Sakurai, Sayaka Okada, Takehiro Ozeki, Rokusuke Yoshikawa, Takaaki Kinoshita, Jiro Yasuda

**Affiliations:** 1 Department of Emerging Infectious Diseases, Institute of Tropical Medicine (NEKKEN), Nagasaki University, Nagasaki, Japan; 2 Department of Emerging Infectious Diseases, National Research Center for the Control and Prevention of Infectious Diseases (CCPID), Nagasaki University, Nagasaki, Japan; 3 School of Tropical Medicine and Global Health, Nagasaki University, Nagasaki, Japan; 4 Graduate School of Biomedical Sciences, Nagasaki University, Nagasaki, Japan; University of Michigan, Ann Arbor, Michigan, USA

**Keywords:** SARS-CoV-2, Omicron, adaptation, spike, host cell entry

## Abstract

The severe acute respiratory syndrome coronavirus 2 (SARS-CoV-2) Omicron variant exhibits high transmissibility with a strong immune escape ability and causes frequent large-scale global infections by producing predominant subvariants. Here, using human upper/lower airway and intestinal cells, we examined the previously dominant BA.1–BA.5 and BA.2.75 subvariants, together with the recently emerged XBB/BQ lineages, in comparison to the former Delta variant. We observed a tendency for each virus to demonstrate higher growth capability than the previously dominant subvariants. Unlike human bronchial and intestinal cells, nasal epithelial cells accommodated the efficient entry of certain Omicron subvariants, similar to the Delta variant. In contrast to the Delta’s reliance on cell-surface transmembrane protease serine 2, all tested Omicron variants depended on endosomal cathepsin L. Moreover, S1/S2 cleavage of early Omicron spikes was less efficient, whereas recent viruses exhibit improved cleavage efficacy. Our results show that the Omicron variant progressively adapts to human cells through continuous endosome-mediated host cell entry.

**IMPORTANCE:**

SARS-CoV-2, the causative agent of coronavirus disease 2019, has evolved into a number of variants/subvariants, which have generated multiple global waves of infection. In order to monitor/predict virological features of emerging variants and determine appropriate strategies for anti-viral development, understanding conserved or altered features of evolving SARS-CoV-2 is important. In this study, we addressed previously or recently predominant Omicron subvariants and demonstrated the gradual adaptation to human cells. The host cell entry route, which was altered from the former Delta variant, was conserved among all tested Omicron subvariants. Collectively, this study revealed both changing and maintained features of SARS-CoV-2 during the Omicron variant evolution.

## INTRODUCTION

Severe acute respiratory syndrome coronavirus 2 (SARS-CoV-2), the causative agent of coronavirus disease 2019 (COVID-19), first appeared in China in 2019 and quickly spread worldwide. During this global pandemic, a number of variants/subvariants have emerged and have been transmitted among humans and animals (1). Starting with prototypic viruses, multiple worldwide infection waves have been created using a series of dominant variants: Alpha, Beta, Gamma, and Delta. From late 2021 to early 2022, the Delta variant was replaced by Omicron, which originally emerged in South Africa (2). Due to its high transmission rate and strong immune evasion ability, the first Omicron variant, BA.1, rapidly generated a global wave of infection, even among the vaccinated or previously infected population (3–5). Since then, numerous Omicron subvariants have emerged and circulated across countries, including the previously dominant BA.1, BA.2, and BA.5, followed by diverse subvariants represented by the XBB and BQ lineages (6). As SARS-CoV-2 continuously evolves and adapts its viral fitness and transmission rate in hosts, it is still worth addressing the virological characteristics of emerging variants.

SARS-CoV-2 has accumulated a number of mutations in its genome during viral evolution. In particular, amino acid changes in viral spike proteins have been extensively studied because of their significant impact on viral infectivity, transmissibility, pathogenicity and antigenicity (7). The SARS-CoV-2 spike protein is expressed on the surface of viral particles and catalyzes host cell entry (8, 9). After being synthesized as a large protein, spike protein is cleaved by the host furin, a ubiquitously expressed pro-protein convertase, to generate the S1 and S2 subunits (10, 11). These non-covalently associate with each other and form a homotrimer on the surface of viral particles (8, 12–14). Once a newly released virus reaches the next target cell, the S1–S2 complex binds to the angiotensin-converting enzyme 2 (ACE2) receptor and undergoes further proteolytic cleavage by either the host transmembrane protease serine 2 (TMPRSS2) or endosomal cathepsin L, leading to membrane fusion on the cell surface or in endosomes, respectively (9, 15, 16).

SARS-CoV-2 has a high affinity for lung/bronchial cells in the human lower airway and therefore shows high pathogenicity in a number of infected individuals (17). This is clear for the Delta variant, which often causes severe pneumonia and involves a high risk of hospital admission and death in infected individuals (18). In contrast, the Omicron variant exhibits decreased affinity for lower airway cells, while possessing high affinity for upper airway cells, such as nasal epithelial cells (19–21). This virological feature contributes significantly to a higher transmission rate between hosts and causes larger scales of global infection by Omicron variants (22). SARS-CoV-2 infection has also been detected in human non-respiratory tissues such as the heart, kidney, and intestines (23, 24). Certain cell subpopulations in these tissues have been experimentally proven to accommodate viral replication (25–27). This tissue/cell tropism might contribute to a variety of COVID-19 symptoms, as well as post-acute sequelae of the disease, termed “long COVID,” which affects multiple organ systems (28). Specifically, in a number of patients, viral antigens and/or RNA can be detected in gastrointestinal biopsies several months after diagnosis, suggesting the role of the intestine as both an active replication site and a persistent reservoir of the virus (29, 30). As SARS-CoV-2 has modulated its affinity for host tissues during evolution, multiple tissues and cell types should be examined to characterize its replication potential in the host.

In this study, we investigated the viral fitness and cell entry efficacy of the major Omicron subvariants and compared these with the Delta variant using multiple human cell lines derived from the lower and upper airways and the intestinal tract. To characterize the viral spike protein and host cell entry, the proteolytic cleavage pattern of the spike proteins and sensitivity to protease inhibitors were assessed for all viruses. This comprehensive study revealed that the Omicron variant progressively adapts to human cells by adjusting virological features, although these are still different from the Delta variant.

## RESULTS

### Growth kinetics of previously dominant SARS-CoV-2 Omicron subvariants

**Fig 1:**
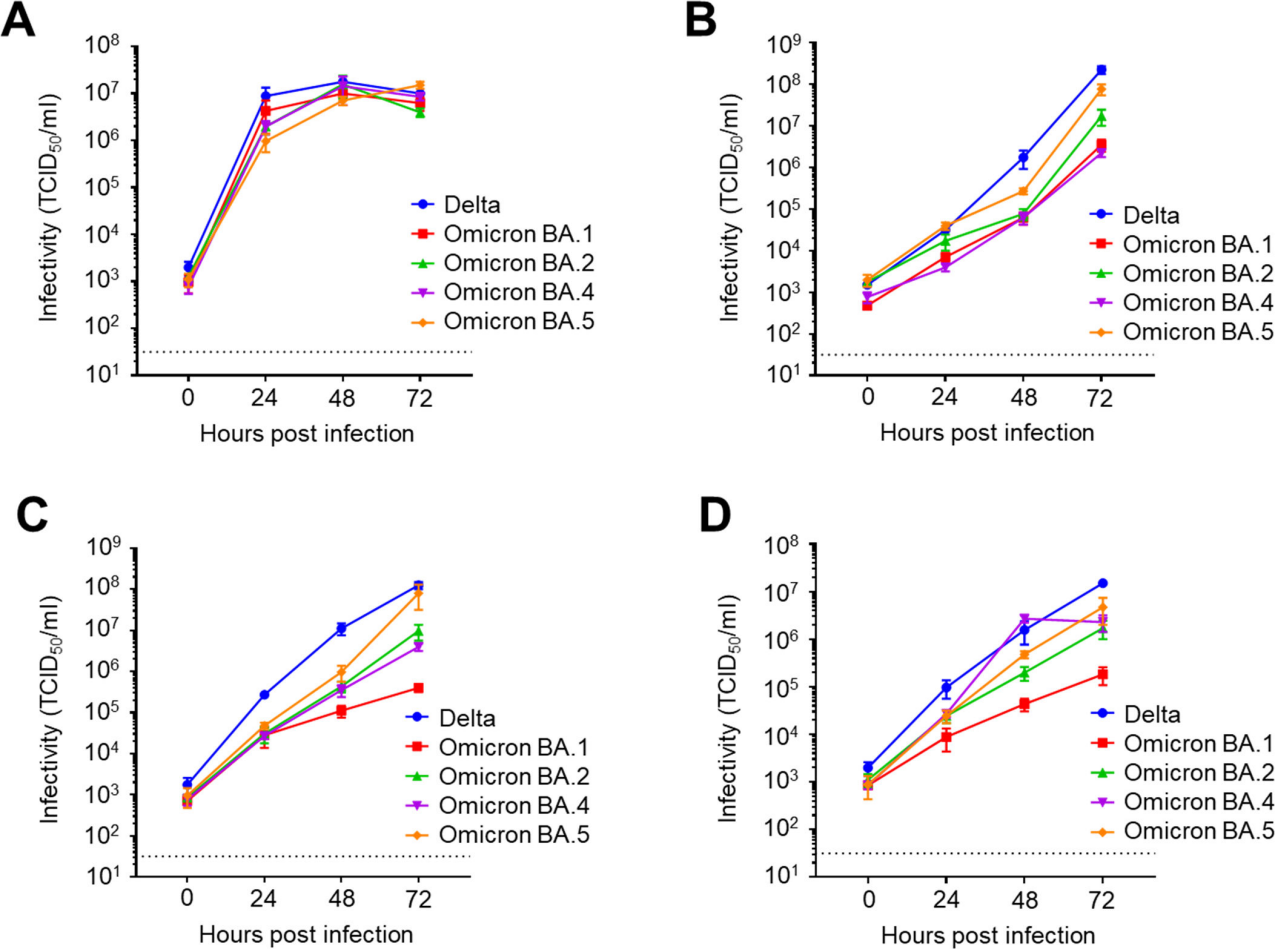
Growth kinetics of SARS-CoV-2 early Omicron subvariants. VeroE6 (**A**), Calu-3 (**B**), Caco-2 (**C**), and RPMI2650 + hACE2 (**D**) cells were infected with SARS-CoV-2 Delta, Omicron BA.1, Omicron BA.2, Omicron BA.4, or Omicron BA.5, at an multiplicity of infection of 0.1. After 0, 24, 48, and 72 h of incubation, the culture supernatant was collected and subjected to TCID_50_ assays using VeroE6/TMPRSS2 cells to determine the viral titers (data presented as mean ± SD, *n* = 3). Each data set is representative of at least two independent experiments. TCID_50_, tissue culture infectious dose.

First, we compared the replication capacities of the Omicron subvariants BA.1, BA.2, BA.4, and BA.5, which had previously circulated worldwide as the first generation of the Omicron variant, as well as the Delta variant. In VeroE6 cells, all tested viruses replicated relatively quickly, which is consistent with existing reports, corroborating the reliability of our assays (Fig. 1A) (21). However, the titers of the released viruses reached a plateau at 48 h after challenge, possibly due to the cytopathic effect, which was evident in the cells. Next, we examined the replication kinetics in human cell lines originating from different tissues. In Calu-3 cells derived from human bronchial tubes, the Omicron subvariants showed decreased replication capacity than the Delta variant, which may be related to their different pathogenicity in humans and animals (Fig. 1B). We also observed that BA.5 replicated more efficiently than other Omicron subvariants, indicating that the virus evolved to acquire a higher growth capacity in human cells. In Caco-2 cells derived from the human intestine, BA.5 again displayed the higher replication efficiency among the tested Omicron subvariants, although all exhibited reduced replication capacity compared to that of the Delta variant (Fig. 1C). Next, to evaluate viral fitness in human upper airway cells, we established an RPMI2650 + hACE2 cell line, which was derived from human nasal RPMI2650 cells and modulated to stably express the human ACE2 protein to be highly sensitive to SARS-CoV-2 replication and entry (Fig. S1). We observed that in this nasal epithelial cell line, all of the tested SARS-CoV-2 variants/subvariants replicated with high efficiency (Fig. 1D), with BA.5 demonstrating the highest replication efficacy among the Omicron subvariants. These results suggest that BA.5 replicates more efficiently than the former Omicron subvariants, including the parental BA.2 in multiple human tissue cells. It is also worth noting that, in contrast to VeroE6 cells, none of the tested human cells showed obvious cytopathic effects and therefore accommodated the constant production of infectious progeny virions without causing virus replication to reach a plateau.

### Growth kinetics of previously emerged SARS-CoV-2 Omicron subvariant BA.2.75

**Fig 2:**
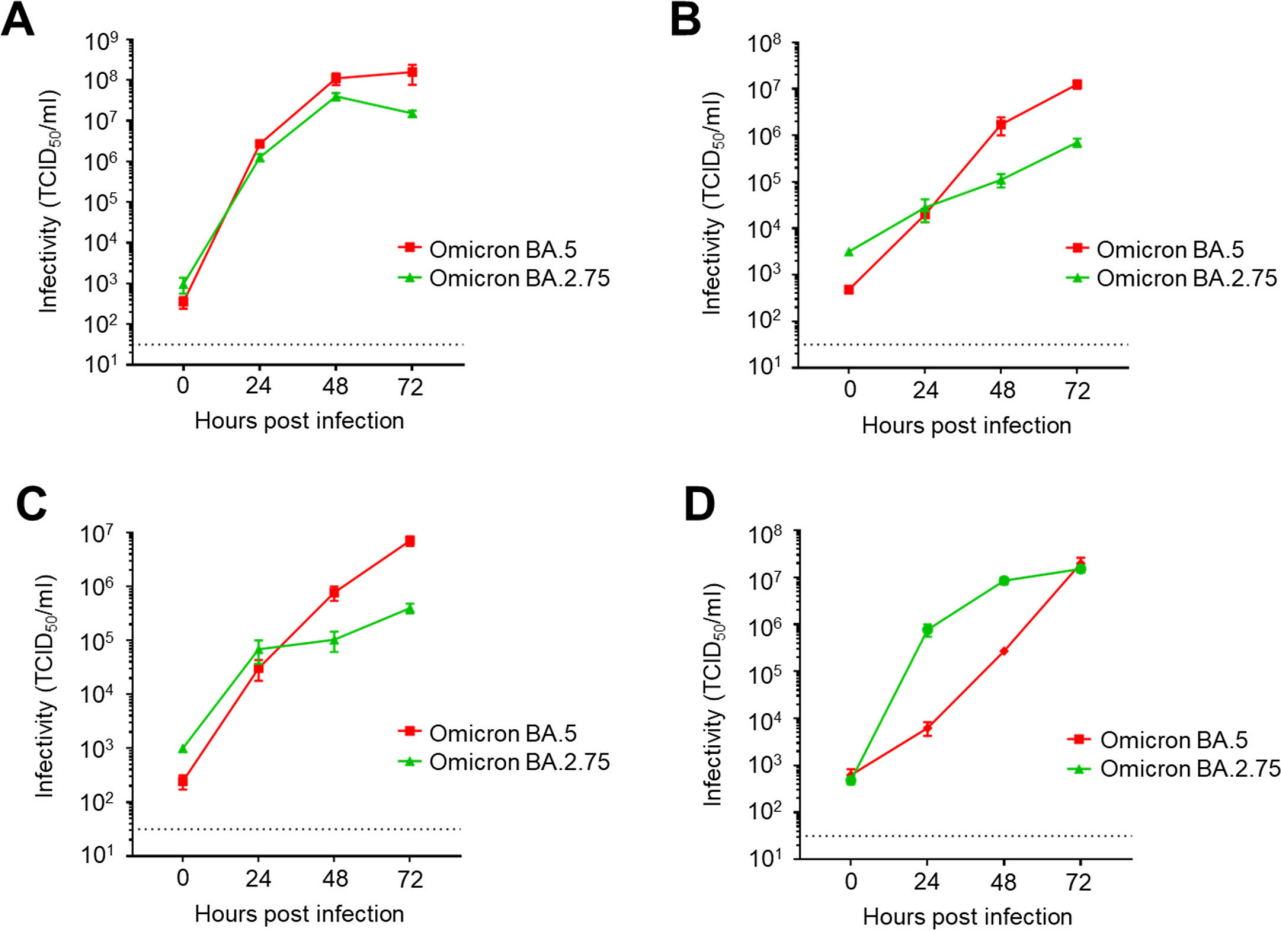
Growth kinetics of the SARS-CoV-2 Omicron BA.2.75 subvariant. VeroE6 (**A**), Calu-3 (**B**), Caco-2 (**C**), and RPMI2650 + hACE2 (**D**) cells were infected with SARS-CoV-2 Omicron BA.5 or Omicron BA.2.75 at a multiplicity of infection of 0.1. After 0, 24, 48, and 72 h of incubation, the culture supernatant was collected and subjected to TCID_50_ assays using VeroE6/TMPRSS2 cells to determine the viral titers (data presented as mean ± SD, *n* = 3). Each data set is representative of at least two independent experiments.

Similar to BA.5, BA.2.75 is a BA.2 descendant and was considered for a time as the most concerning variant because of its strong affinity with the ACE2 receptor (31). Therefore, we investigated the replication capacity of BA.2.75 in comparison with BA.5. Similar to other Omicron subvariants, BA.2.75 efficiently replicated in VeroE6 cells (Fig. 2A). In human bronchial Calu-3 cells and the intestinal Caco-2 cells, BA.2.75 exhibited a lower replication capacity than BA.5 (Fig. 2B and C). In contrast, BA.2.75 displayed faster growth than BA.5 in human nasal RPMI2650 + hACE2 cells, especially at the early time points (Fig. 2D). Therefore, BA.2.75 appears to specifically have a high replication capacity in cells derived from the human upper respiratory tract.

### Growth kinetics of SARS-CoV-2 Omicron BQ.1 and XBB lineages

**Fig 3:**
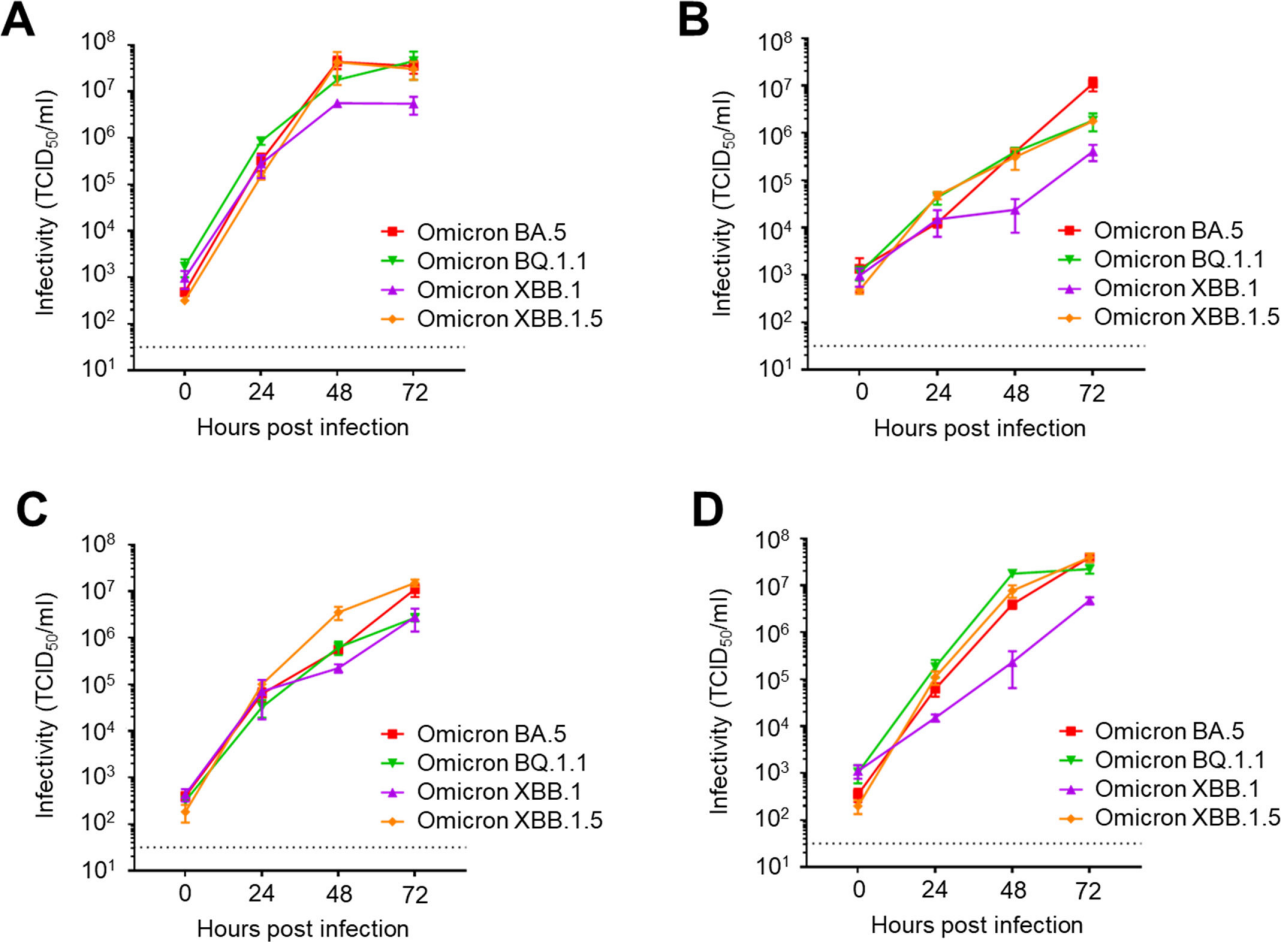
Growth kinetics of the SARS-CoV-2 BQ.1 and XBB lineages. VeroE6 (**A**), Calu-3 (**B**), Caco-2 (**C**), and RPMI2650 + hACE2 (**D**) cells were infected with SARS-CoV-2 Omicron BA.5, Omicron BQ.1.1, Omicron XBB.1, or Omicron XBB.1.5, at a multiplicity of infection of 0.1. After 0, 24, 48, and 72 h of incubation, the culture supernatant was collected and subjected to TCID_50_ assays using VeroE6/TMPRSS2 cells to determine the viral titers (data presented as mean ± SD, *n* = 3). Each data set is representative of at least two independent experiments.

After the global wave of BA.5, multiple local subvariants, including major BQ.1, and XBB lineages, which are descendants of BA.5 and BA.2 respectively, have become prevalent in tandem. Among these, BQ.1.1, XBB.1, and XBB.1.5 (=the progeny of XBB.1) emerged as local dominant variants and then spread globally. Because of the importance of these emerged subvariants in public health, we examined their growth kinetics in multiple cell types. In VeroE6 cells, all of BQ.1.1, XBB.1, and XBB.1.5 demonstrated efficient replication, although XBB.1 showed lesser growth capacity than others (Fig. 3A). In human bronchial Calu-3 cells and nasal RPMI2650 + hACE2 cells, BQ.1.1 and XBB.1.5 showed high growth capacity comparable to BA.5, while XBB.1 replicated at a slower rate than the others (Fig. 3B and D). In human intestinal Caco-2 cells, XBB.1.5 exhibited the fastest replication among the tested Omicron subvariants (Fig. 3C). Overall, XBB.1.5 demonstrated the ability to replicate highly efficiently in a diverse range of cells, suggesting the adaptation of recently emerged subvariants to multiple human tissue cells.

### Comparative analysis of spike-dependent host cell entry of SARS-CoV-2 Omicron subvariants

**Fig 4:**
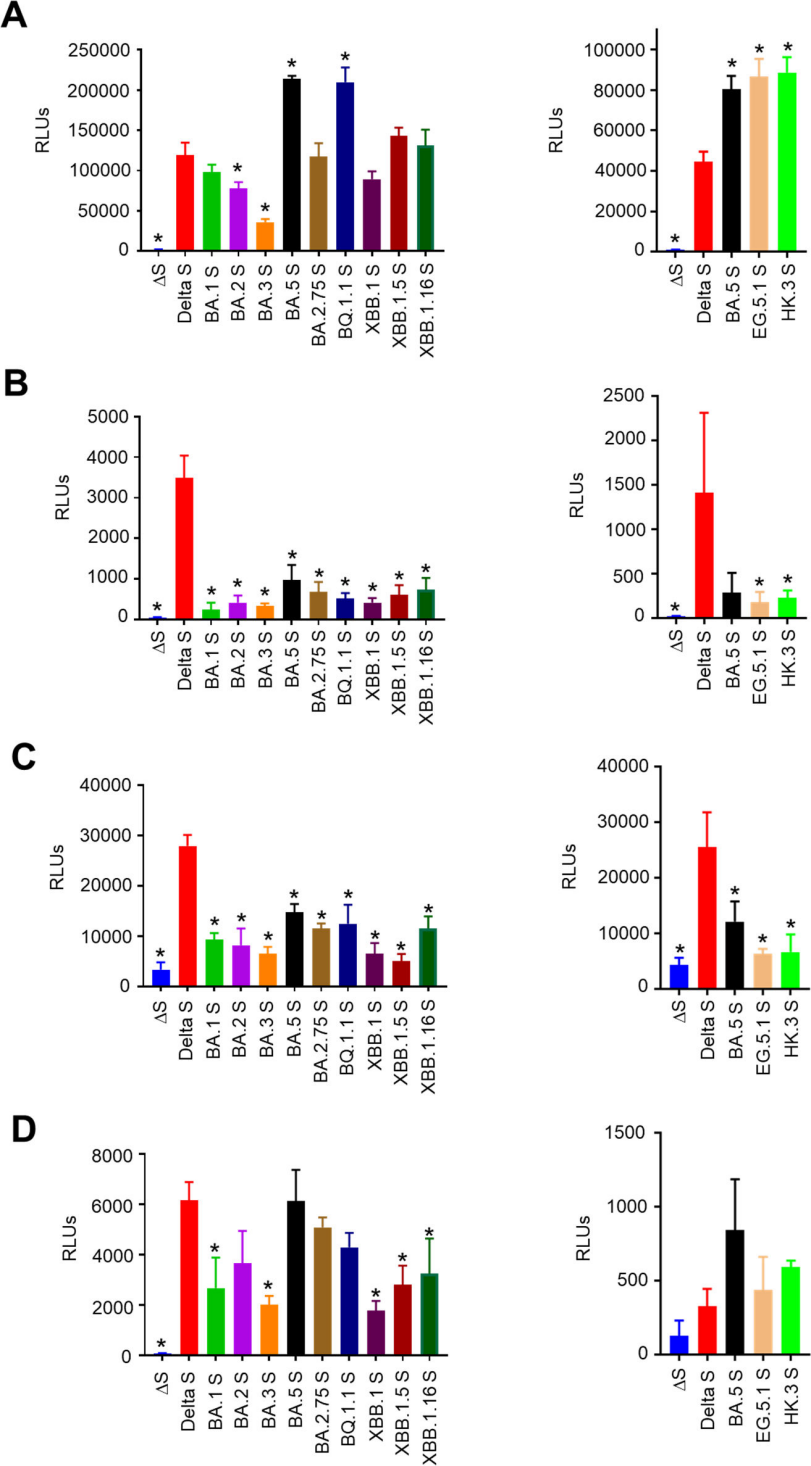
Host cell entry efficacy of SARS-CoV-2 Omicron subvariants. VeroE6 (**A**), Calu-3 (**B**), Caco-2 (**C**), and RPMI2650 + hACE2 (**D**) cells were infected with VSV pseudotyped with spike proteins derived from each SARS-CoV-2 Omicron subvariant (left panels: BA.1, BA.2, BA.3, BA.5, BA.2.75, BQ.1.1, XBB.1, XBB.1.5, and XBB.1.16; right panels: BA.5, EG.5.1, and HK.3) or the Delta variant. After 1 day of incubation, luciferase activity in infected cells was measured in relative light units (RLUs) to evaluate the virus infectivity (data presented as mean ± SD, *n* = 3). Each data set is representative of at least two independent experiments. One-way analysis of variance (*P < 0.05) was used, compared to Delta S.

The viral fitness of SARS-CoV-2 is considered to largely depend on the function of spike proteins, which catalyze viral entry into host cells (32). Similar to live SARS-CoV-2, VeroE6 cells were highly sensitive to all pseudotyped viruses bearing SARS-CoV-2 spike proteins. Especially, viruses with spike proteins derived from BA.5 and its descendant, BQ.1.1., showed a relatively higher entry efficacy (Fig. 4A). In human Calu-3 and Caco-2 cells, viruses bearing spike proteins derived from Omicron variants showed significantly lower entry efficacy than the Delta variant, consistent with their lower pathogenicity (Fig. 4B and C). In contrast, certain Omicron subvariants, such as BA.5, BA. 2.75, and BQ.1.1, as well as recent EG.5.1 and HK.3, exhibited comparably high entry efficacy to that of the Delta variant in human nasal RPMI2650 + ACE2 cells, suggesting their high affinity with nasal epithelial cells and, therefore, high transmission capacity (Fig. 4D). Of note, BA.5, BA. 2.75, and BQ.1.1, which were reported to have a high affinity with the ACE2 receptor, showed higher entry efficacy than that of other Omicron subvariants, indicating the role of ACE2-binding affinity in Omicron evolution in terms of host cell entry (33).

### Characterization of cell entry routes of SARS-CoV-2 Omicron subvariants

**Fig 5:**
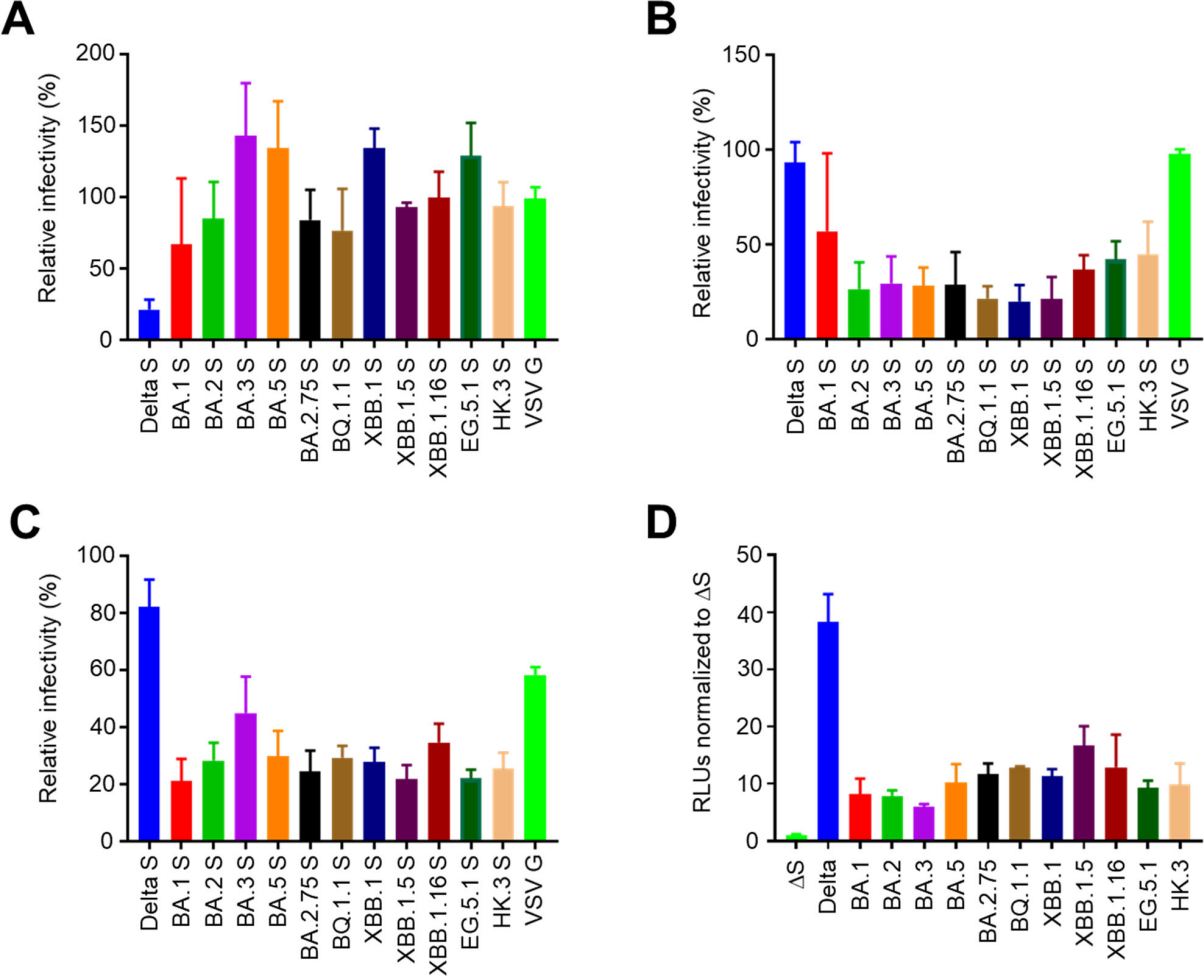
Cell entry route characteristics of SARS-CoV-2 Omicron subvariants. (A–C) Caco-2 cells were pretreated with 10 µM of nafamostat (**A**), E64d (**B**), or chloroquine (**C**) and infected with VSV pseudotyped with spike proteins derived from each SARS-CoV-2 Omicron subvariant (left panels: BA.1, BA.2, BA.3, BA.5, and BA.2.75; right panels: BQ.1.1, XBB.1, XBB.1.5, XBB.1.16, EG.5.1, and HK.3), or the Delta variant, or glycoproteins derived from VSV in the presence of each compound. After 1 day of incubation, luciferase activity in infected cells was measured to evaluate the virus infectivity (data presented as mean ± SD, *n* = 3). Each data set is representative of three independent experiments. (**D**) Spike-based fusion assay was performed using 293T cells transiently expressing spike proteins derived from each SARS-CoV-2 Omicron subvariant or the Delta variant, and Caco-2 cells transfected with a pT7EMCV-Luc plasmid. Twenty-four hours after mixing both cells, luciferase activity was measured to evaluate the fusion activity (data presented as mean ± SD, *n* = 3). Each data set is representative of three independent experiments.

During host cell entry, the SARS-CoV-2 spike protein is proteolytically cleaved by either cell-surface TMPRSS2 or endosomal cathepsin L, and the preference for the host protease depends on the variant and host cell types (34). Using Caco-2 cells endogenously expressing both TMPRSS2 and cathepsin L, we demonstrated that the TMPRSS2 inhibitor, nafamostat, potently suppressed the entry of the Delta variant but not the entry of either Omicron subvariant (Fig. 5A). In contrast, the cathepsin inhibitor E64d efficiently inhibited the entry of all Omicron subvariants but did not inhibit that of the Delta variant (Fig. 5B). Consistently, all Omicron subvariants, but not the Delta variant, were sensitive to chloroquine, an endosomal maturation inhibitor (Fig. 5C). We did not note any significant difference among the Omicron subvariants regarding their sensitivity to inhibitors. Taken together, these data indicate that the Delta variant preferably utilizes the cell-surface TMPRSS2 for the entry to host cells, whereas the Omicron variant uses endosomal cathepsin L for this purpose, which is a conserved property among all tested Omicron subvariants.

To further support this claim, we conducted a spike-mediated fusion assay, which mainly quantifies the TMPRSS2-dependent fusion events occurring on the cell surface, but minimally reflects the cathepsin-dependent fusion events in endosomes. Following this approach, we observed that all Omicron subvariants displayed significantly lower fusion activity relative to the Delta variant, confirming the preference of Delta and Omicron variants for the cell-surface TMPRSS2 and endosomal cathepsin L, respectively (Fig. 5D). No significant differences in fusion activity were detected among the Omicron subvariants. Overall, these results suggest that the spike protein of the Delta variant exhibits strong fusion activity on the cell surface, while those of all tested Omicron subvariants likely lost their activities, resulting in their dependence on endosomal routes for entry.

### Characterization of proteolysis of spike proteins derived from SARS-CoV-2 Omicron subvariants

**Fig 6:**
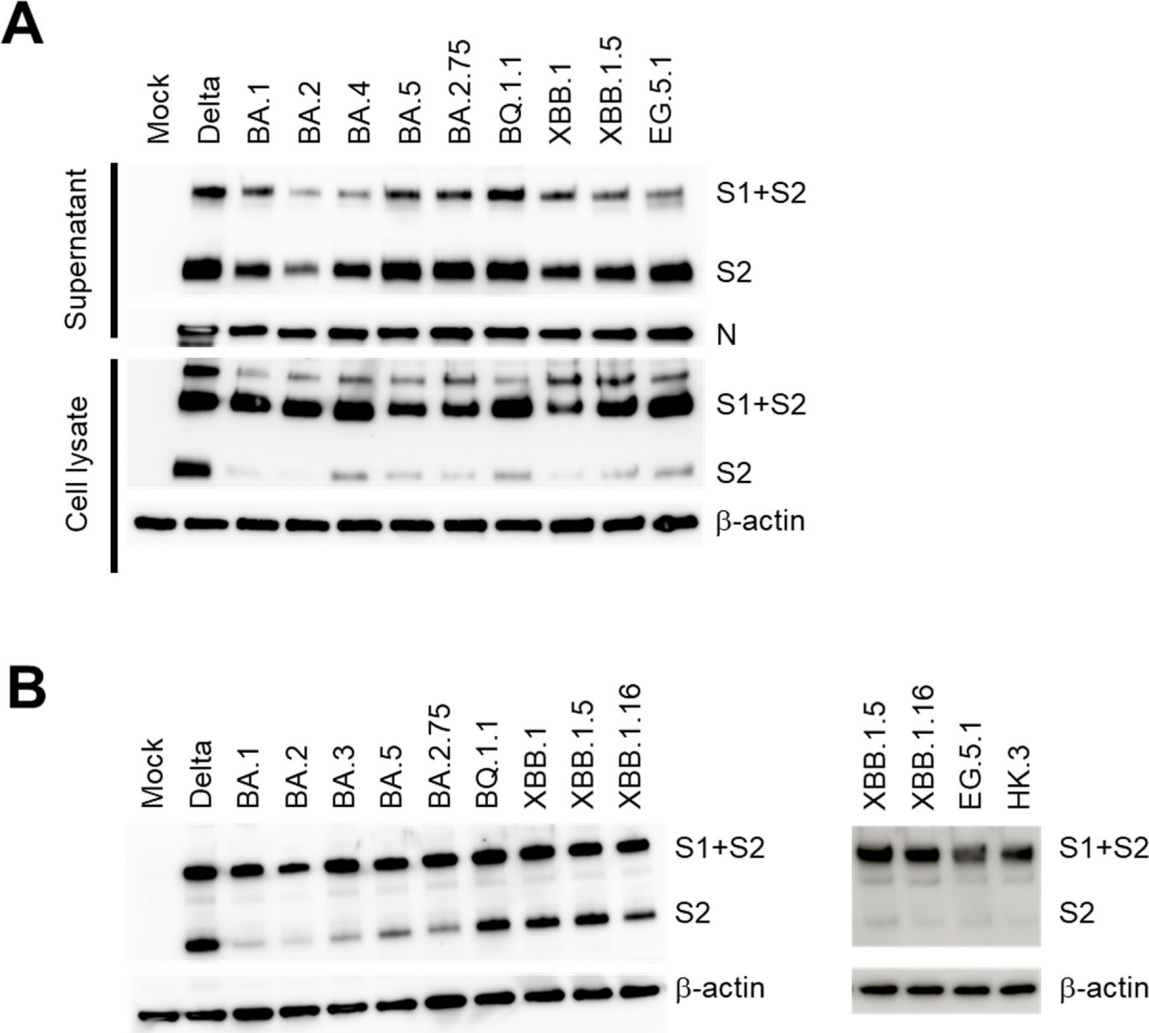
Proteolytic cleavage patterns of spike proteins derived from SARS-CoV-2 Omicron subvariants. (**A**) VeroE6/TMPRSS2 cells were infected with each SARS-CoV-2 Omicron subvariant or the Delta variant. At day 1 post-infection, the supernatant was collected and concentrated by ultracentrifugation, and cell lysates were also prepared. Both types of samples were subjected to SDS-PAGE and immunoblotting to detect viral spike proteins. S1/S2 and S2 indicate full-length (uncleaved) spike protein and cleaved S2 protein, respectively. The N protein of SARS-CoV-2 and β-actin were used as loading controls. The data set is representative of at least two independent experiments. (**B**) 293T cells were transfected with a plasmid expressing spike protein derived from each SARS-CoV-2 Omicron subvariant (left panels: BA.1, BA.2, BA.3, BA.5, BA.2.75, BQ.1.1, XBB.1, XBB.1.5, and XBB.1.16; right panels: XBB.1.5, XBB.1.16, EG.5.1, and HK.3) or the Delta variant. At day 2 post-transfection, cell lysates were prepared. The samples were subjected to SDS-PAGE and immunoblotting to detect viral spike proteins. The S1/S2 and S2 indicate the full-length (uncleaved) and cleaved S2 spike protein, respectively. β-actin was used as a loading control. The data set is representative of at least two independent experiments.

The SARS-CoV-2 spike protein has another cleavage site at the S1/S2 junction, which is recognized by the host furin or similar enzymes to generate S1 and S2 proteins during the biogenesis of the viral protein (34). The spike protein of the Delta variant was efficiently cleaved in virus-infected cells, and the cleaved active form was highly expressed in the released virus particles (Fig. 6A). Among the Omicron variants, recent subvariants including BQ.1 and XBB lineages showed higher levels of cleaved spike proteins than early subvariants. This cleavage pattern was also confirmed by detecting the proteins in human cells exogenously expressing spike proteins (Fig. 6B). This is consistent with the elevated growth rates of these Omicron subvariants and their high cell entry efficacies, as shown in Fig. 3 and 4.

## DISCUSSION

In this study, we conducted comprehensive analyses of SARS-CoV-2 Omicron subvariants by considering the order of appearance and identified differences in their viral fitness *in vitro*. After the initial Omicron subvariant BA.1 displaced the Delta variant as a predominant virus in early 2021, other Omicron subvariants BA.2 and BA.5 emerged and circulated globally in succession (6). Consistent with this epidemiological tendency, our results demonstrated a gradual increase in the viral fitness of these early Omicron subvariants, with BA.5 showing the highest replication capacity in human cells. Another Omicron subvariant, BA.2.75, emerged and became predominant in some regions during a global wave of BA.5. Our study indicated its limited growth capacity compared to BA.5. Consistent with the information in public database, the phylogenetic analysis of the strains tested in this study indicated that BA.2.75 belonged to a phylogenetically different group from BA.5 (Fig. S2A). Considering that their circulation time was overlapped (Fig. S2B) and BA.2.75 could not widely outcompete BA.5, BA.2.75 is not superior to BA.5 in terms of the adaptation to human cells, which was also supported by our results (Fig. S2B). During the worldwide circulation of BA.5, the Omicron variant became highly diversified to the extent of an amply termed “variant soup,” from which two major lineages, BQ.1 and XBB, emerged (35). Among the BQ.1 lineage, which was generated as a result of convergent evolution from BA.5, BQ.1.1 became the predominant strain in many Western countries in late 2022 and early 2023 (36). In our study, BQ.1.1 demonstrated comparable or slightly higher replication efficacy than BA.5 in all tested human cells because of its adaptation to human cells, as previously reported (37). In contrast, the XBB lineage, which emerged in India in mid-August 2022, was generated as a result of a recombination event between two BA.2 descendants: BJ.1 and BM.1.1.1 (a progeny of BA.2.75) (38). This variant further evolved to produce XBB.1 and then XBB.1.5, which was predominant in the United States by outcompeting BQ.1.1 in early 2023, before spreading internationally (39). The XBB.1 exhibited weak viral fitness compared to BA.5, likely owing to its immature adaptation to human cells after the recombination event for the lineage. In contrast, its descendant, XBB.1.5, exhibited a significantly higher growth capacity than XBB.1 and comparable or slightly higher replication efficacy than BA.5 and BQ.1.1 in all tested human cells, suggesting strong adaptation of the subvariant to human cells. Overall, the Omicron variant has evolved to increase infectivity and outcompete other predominant subvariants, resulting in the progressive adaptation of viruses to humans. Our work also indicated that growth capacity in host cells is still a major determinant for becoming a predominant virus among other Omicron variants.

In contrast to many other respiratory viruses, SARS-CoV-2 has an ability to infect multiple human tissues, which may cause a variety of symptoms in patients with COVID-19 (40). Therefore, we addressed the viral fitness and entry efficacy in cells derived from the human intestine and nasal cavity, which express SARS-CoV-2 entry factors, in addition to the well-studied lung/bronchial cells (41). Specifically, certain cell types, such as intestinal enterocytes, highly express the viral entry receptor ACE2 and have been suggested to be attractive replication sites for SARS-CoV-2 (25). In our study using Caco-2 cells, which are enterocyte-like cells that express both ACE2 and the host protease TMPRSS2/cathepsin L, most Omicron subvariants showed high growth capacity in the human colorectal cell line (42, 43). Similar to human bronchial Calu-3 cells, the overall viral fitness of Omicron variants was lower than that of the Delta variant in Caco-2 cells, whereas certain recent subvariants exhibited high growth capacities, reaching that of the Delta variant. Therefore, gastrointestinal symptoms should be monitored in patients to identify newly emerging variants. We also examined a cell type derived from the human nasal epithelium, which is the initial entry portal for human coronaviruses and plays a crucial role in viral transmission (44). Unfortunately, to our knowledge, there is no human nasal epithelial cell line permissive to SARS-CoV-2 replication, including the commonly used RPMI2650 cells, although certain cell types in human nasal cavity express ACE2 and are permissive to SARS-CoV-2 infection (41, 42, 44). Therefore, we established RPMI2650 cells that highly express human ACE2 and demonstrated that SARS-CoV-2 efficiently replicated in this cell line, suggesting that it is a useful tool for coronavirus research. Using this cell line, we identified that differences in viral fitness and entry efficacies between the Delta and Omicron variants were smaller than those in other human cells. Surprisingly, BA.2.75 showed unexpectedly high replication and entry capacities into the cell line, suggesting its high affinity for nasal epithelial cells. Considering its high binding affinity for the ACE2 receptor, BA.2.75 and its sublineages may have a high transmission capacity between human populations (33).

During biosynthesis of the S protein in virus-producing cells, the large full-length spike protein is cleaved mainly by the host furin to separate the S1 and S2 subdomains. This property of the spike protein has been considered to contribute to the transmissibility and pathogenicity of SARS-CoV-2 and distinguish it from closely related coronaviruses, including SARS-CoV (45–47). Consistent with previous reports, our study showed that the efficacy of S1/S2 cleavage varied among the Omicron subvariants, although this did not clearly correlate with their fitness *in vitro*. It might be caused by usage of 293T cells and VeroE6 cells, but not Caco-2 cells, Calu-3 cells, and RPMI2650 + hACE2 cells. Unfortunately, due to extremely low transfection and infection efficacy in those cell lines, spike expression could not be efficiently detected in them. Interestingly, variants and subvariants demonstrating higher cleavage efficacy, such as BQ.1.1, and the recent XBB-lineage viruses, contained higher levels of the cleaved spike protein form (S2 protein in Fig. 6) in the produced viral particles. Such viruses should have a higher chance of infecting host cells, even those expressing low levels of ACE2, and therefore may exhibit higher transmissibility between different hosts. Although this hypothesis could not be examined in detail in our study using only cell lines highly expressing ACE2, it nevertheless aligns well with previous reports regarding recently emerged subvariants displaying higher transmissibility by an indirect contact (airborne) route than early Omicron subvariants in animal models (48–50).

During the cell entry, the SARS-CoV-2 spike protein undergoes another proteolytic cleavage at the S2′ site by the host serine–protease TMPRSS2 on the cell surface or the pH-dependent cysteine protease cathepsin L in endosomes. Consistent with findings in the previous studies, our experiments demonstrated that the Delta variant preferably utilized a TMPRSS2-dependent entry, in contrast to the Omicron variant, which predominantly internalized via the cathepsin-dependent route (51, 52). This feature was conserved among all tested Omicron subvariants. Importantly, cathepsin L can cleave multiple sites in the spike protein and therefore may bypass furin-mediated S1/S2 cleavage for the full activation of spike proteins, leading to membrane fusion (53). As spike proteins of Omicron variants exhibit lower S1/S2 cleavage efficacy during spike biogenesis, they depend primarily on host cathepsin activity to achieve successful cell entry. Following endosomal routes, the Omicron variant encountered additional host restriction factors, such as interferon-inducible IFITM2/IFITM3 (54). It has been reported that interferon responses in nasal epithelial cells are delayed at onset, which might contribute to the efficient entry of the Omicron variant into nasal epithelial RPMI2650 + ACE2 cells, compared to lung/bronchial Calu-3 cells (55). In regard to the molecular basis, although the amino acid sequence of the S2′ site and putative cathepsin cleavage sites (not fully determined yet) is conserved between Delta and Omicron variants, a previous study highlighted the C-terminus side of the S2′ site in the spike protein as the determinant of the entry route preferences (54). All of our tested Omicron subvariants had N950D, Q954H, and N969K mutations relative to the Delta variant in the region. All or either of the amino acid changes may be responsible for the dependence of the Omicron variant on processing by endosomal cathepsin L. Because the viral entry route is crucial for several anti-viral compounds to exhibit activity, this characteristic of the Omicron variant should be continuously monitored for future anti-viral development.

## MATERIALS AND METHODS

### Cells

The VeroE6 cells (donated by Dr. Ayato Takada, Hokkaido University, Japan), Calu-3 cells (donated by Dr. Shutoku Matsuyama, National Institute of Infectious Diseases, Japan), Caco-2 cells (donated by Dr. Tetsuya Iida, Osaka University, Japan), and RPMI2650 cells (Cosmo Bio, Japan) were all maintained in Dulbecco’s modified Eagle’s medium (DMEM) supplemented with 10% fetal bovine serum (FBS) and 1% penicillin/streptomycin (PS) solution. The VeroE6/TMPRSS2 cells (VeroE6 cells constitutively expressing human TMPRSS2) were donated by Dr. Shutoku Matsuyama (National Institute of Infectious Diseases, Japan) and were maintained in DMEM supplemented with 10% FBS, 1% PS, and 500 μg/mL of G418 (56). To establish RPMI2650/ACE2 cells (RPMI2650 cells stably expressing human ACE2), the human *ACE2* gene was amplified from the total RNA extract of Calu-3 cells, fused with a FLAG tag at its C-terminus, and cloned into the pCXN2 plasmid to generate pCXN2–hACE2–FLAG. The RPMI2650 cells were transfected with pCXN2–hACE2–FLAG using Lipofectamine 3000 (Thermo Fisher Scientific, Waltham, MA, USA) and selected with 5 μg/mL of puromycin (InvivoGen, San Diego, CA, USA). Established RPMI2650/ACE2 cells were maintained in DMEM supplemented with 10% FBS, 1% PS, and 5 μg/mL of puromycin.

### Propagation and titration of SARS-CoV-2

Clinical isolates of Delta (strain TY11-927, GISAID ID: EPI_ISL_2158617), Omicron BA.1 (strain TY38-873, GISAID ID: EPI_ISL_7418017), Omicron BA.2 (strain TY40-385, GISAID ID: EPI_ISL_9595859), Omicron BA.4 (strain TY41-703, GISAID ID: EPI_ISL_13278440), Omicron BA.5 (strain TY41-702, GISAID ID: EPI_ISL_13241867), Omicron BA.2.75 (strain TY41-716, GISAID ID: EPI_ISL_13969765), Omicron BQ.1.1 (strain TY41-796, GISAID ID: EPI_ISL_15579783), Omicron XBB.1 (strain TY41-795, GISAID ID: EPI_ISL_15669344), and Omicron XBB.1.5 (strain 23–018, GISAID ID: EPI_ISL_16889601) were donated by the National Institute of Infectious Diseases (Japan). Clinical isolates of Omicron HK.3 (strain ET2023) were isolated from a Japanese patient. All variants/subvariants of SARS-CoV-2 were propagated in VeroE6/TMPRSS2 and VeroE6 cells as previously described (57). Culture supernatants were collected 3–5 days after infection, cleared by centrifugation at 2,000 × *g* for 15 min and stored at −80°C until use. The viral titer was determined by a plaque assay using VeroE6 cells. After 1 h of infection, the inoculum was washed and the cells were incubated with 0.7% agarose gel in Minimum Essential Medium (MEM) supplemented with 2% FBS and 1% PS solution for three days. After viral inactivation using 4% paraformaldehyde (PFA) overnight, cells were stained with crystal violet solution. The plaques were counted manually to calculate viral titers. All experiments with replication-competent SARS-CoV-2 were performed in a biosafety level three laboratory at Nagasaki University.

### Growth kinetics analysis of SARS-CoV-2

To evaluate the replication efficacy of each SARS-CoV-2 variant/subvariant, VeroE6, Calu-3, Caco-2, and RPMI2650/ACE2 cells were plated in 12 well plates and infected with each virus at an multiplicity of infection (MOI) of 0.1 for 30 min. After washing out the inoculum, cells were incubated at 37°C, and the supernatant was collected at indicated time points and stored at −80°C until use. The infectious viral titer in the supernatant was quantified as previously described (58). Briefly, VeroE6/TMPRSS2 cells were plated in 96-well plates and cultured in DMEM supplemented with 2% FBS at 37°C until they reached 100% confluence. Each supernatant was serially diluted 10-fold and transferred into a 96 well plates containing VeroE6/TMPRSS2 cells. After at least four days of incubation at 37°C, the cytopathic effects of cells in each well were observed under a microscope, and the median tissue culture infectious dose was calculated.

### Cloning of SARS-CoV-2 S genes

We synthesized codon-optimized *S* genes of SARS-CoV-2 Delta (GISAID ID: EPI_ISL_2158617), Omicron BA.1 (GISAID ID: EPI_ISL_7418017), Omicron BA.2 (EPI_ISL_8559646), Omicron BA.4/BA.5 (GISAID ID: EPI_ISL_13241867), Omicron BA.2.75 (GISAID ID: EPI_ISL_13969765), Omicron BQ.1.1 (GISAID ID: EPI_ISL_15579783), Omicron XBB.1 (GISAID ID: EPI_ISL_15669344), Omicron XBB.1.5 (GISAID ID: EPI_ISL_17194415), Omicron XBB.1.16 (GISAID ID: EPI_ISL_17718585), and Omicron EG.5.1 (GISAID ID: EPI_ISL_17432511), each carrying a 19 amino acid deletion at the C terminus. The *S* genes of SARS-CoV-2 Omicron HK.3 were synthesized by introducing the L455F mutation into the *S* gene of Omicron EG.5.1, using the KOD Plus Mutagenesis kit (TOYOBO, Osaka, Japan). All synthesized *S* genes were inserted into the pCAGGS expression plasmid using In-Fusion HD (Clontech, Mountain View, CA), following the manufacturer’s instructions.

### Production and infection of pseudotyped viruses

We generated the VSV-based pseudotyped virus bearing spike proteins of each SARS-CoV-2 variant/subvariant (VSVΔG–SARS2-S) using a recombinant VSV where the *VSV-G* gene was substituted for a firefly luciferase reporter gene (VSVΔG–VSV-G) as previously described (59). The 293T cells were transfected with a pCAGGS plasmid encoding each SARS-CoV-2 *S* gene using the TransIT LT1 transfection reagent (Mirus, Madison, WI, USA). One day after transfection, the cells were infected with VSVΔG–VSV-G for 1 h. The supernatant was harvested 1 d after infection, cleared by centrifugation at 2,000 × *g* for 15 min, and stored at −80°C until use. As a control for contamination of the inoculating virus, the cells were transfected with a pCAGGS plasmid and challenged with VSVΔG–VSV-G for 1 h, followed by collection of the culture supernatant 1 day post-infection. To normalize the amount of pseudotyped virus for subsequent experiments, the relative amount of virus in each supernatant was quantified by reverse transcription quantitative PCR using a primer set specific to the *VSV-M* gene, as described in earlier work (60).

To address the infectivity of VSVΔG–SARS2-S in multiple cell types, VeroE6, Calu-3, Caco-2, and RPMI2650/ACE2 cells were plated in each 96-well plate and challenged with each pseudotyped VSV with incubation at 37°C. After 1 day, the cells were lysed and luciferase activity was measured using Steady-Glo Luciferase Assay System (Promega, Madison, WI, USA) and a SpectraMax iD5 microplate reader (Molecular Devices, San Jose, CA, USA).

To evaluate the effects of compounds on pseudotyped VSV infection, Caco-2 cells were plated in 96-well plates and incubated with 10 µM of nafamostat (Selleck Chemicals, Houston, TX, USA), E64d (Selleck Chemicals) or chloroquine (Sigma-Aldrich, St. Louis, MO) for 1 h. Then, cells were challenged with each pseudotyped VSV and incubated in the presence of compounds at 37°C for 24 h. The next day, cells were lysed, and luciferase activity was measured as described above.

### SARS-CoV-2 spike-based fusion assay

We evaluated the membrane fusion activity of the SARS-CoV-2 spike proteins as follows: 293T cells were co-transfected with a pCAGGS plasmid encoding each SARS-CoV-2 *S* gene and a T7 polymerase expression plasmid using the TransIT LT1 transfection reagent. In parallel, Caco-2 cells were transfected with a pT7EMCV-Luc plasmid (donated by Dr. Takayuki Miyazawa, Kyoto University, Japan) encoding a firefly luciferase reporter gene whose expression is regulated by the T7 promoter, using the Lipofectamine 3000 transfection reagent. One day after transfection, both transfected 293T and Caco-2 cells were detached, mixed at a 5:4 ratio, and replated into 96-well plate. After 24 h, the cells were lysed, and luciferase activity was measured using a SpectraMax iD5 microplate reader.

### Detection of SARS-CoV-2 spike expression

Expression of SARS-CoV-2 spike proteins was examined in lysates of cells infected with SARS-CoV-2 or transfected with spike-expressing plasmids as well as culture supernatants containing SARS-CoV-2. For transfected cells, 293T cells were transfected with a pCAGGS plasmid encoding each SARS-CoV-2 *S* gene using the TransIT LT1 transfection reagent. Two days after transfection, cells were collected and resuspended in PBS. For spike protein detection in infected cells, VeroE6/TMPRSS2 cells were infected with each variant/subvariant of SARS-CoV-2 at an MOI of 0.1. After 24 h of incubation, the infected cells were lysed in Radioimmunoprecipitation Assay (RIPA) buffer [150-mM NaCl, 1.0% Triton X-100, 0.5% sodium deoxycholate, 0.1% SDS, and 50-mM Tris (pH 8.0)]. To detect spike proteins in the released viruses, VeroE6/TMPRSS2 cells were infected with each SARS-CoV-2 variant/subvariant at an MOI of 0.1. After incubation for 48 h, the supernatant was collected, concentrated by ultracentrifugation at 55,000 rpm for 1 h, and resuspended in PBS. Then, the cell lysates and concentrated supernatant samples were incubated with sample buffer solution (Nacalai Tesque, Kyoto Japan) at 95°C for 10 min and subjected to SDS-PAGE in a 4%–20% gradient polyacrylamide gel (Bio-Rad Laboratories, Hercules, CA, USA). The resolved proteins were transferred onto a 0.2-µm polyvinylidene fluoride membrane using the Trans-Blot Turbo Transfer System (Bio-Rad Laboratories). The blots were blocked with 5% skim milk in Tris-buffered saline–Tween 20 and incubated at 4°C overnight with each of the following primary antibodies: anti-SARS-CoV-2 spike [1A9] (GeneTex, Irvine, CA, USA), anti-SARS-CoV-2 nucleocapsid [7E1B] (Bioss Antibodies, Woburn, MA, USA), anti-VSV-M [23H12] (Kerafast, Boston, MA, USA), and anti-β actin [AC-15] (Sigma-Aldrich). The blots were then incubated at room temperature for 1 h with anti-mouse IgG–HRP antibody (Sigma-Aldrich), followed by protein detection using ECL Prime (GE Healthcare, Chicago, IL, USA) according to the manufacturer’s instructions. The protein bands were visualized using an image analyzer (LAS-4000 Mini, GE Healthcare).

### Phylogenetic analysis

To infer the phylogeny of tested SARS-CoV-2 variants/subvariants, *S* gene sequences of the viruses except for the Omicron HK.3 subvariant were obtained from the GISAID website. A sequence of *S* gene derived from HK.3 virus (strain ET2023) was determined as described previously (61). Then, a maximum‐likelihood tree was generated using MEGA version 11 software under the condition of a best‐fit substitution model T92 + G.

## SUPPLEMENTAL MATERIAL

Supplemental Figures msphere.00338-24-s0001.pdf Fig. S1 and S2.

ASM does not own the copyrights to Supplemental Material that may be linked to, or accessed through, an article. The authors have granted ASM a non-exclusive, world-wide license to publish the Supplemental Material files. Please contact the corresponding author directly for reuse.
