# Supplementary material for: SARS-CoV-2 Omicron subvariants progressively adapt to human cells with altered host cell entry
Source: mSphere. 2024 Aug 27;9(9):e00338-24. doi: 10.1128/msphere.00338-24 (PMC11423564; doi:10.1128/msphere.00338-24)
Supplement: Supplemental Figures — Fig. S1 and S2. [file msphere.00338-24-s0001.pdf]

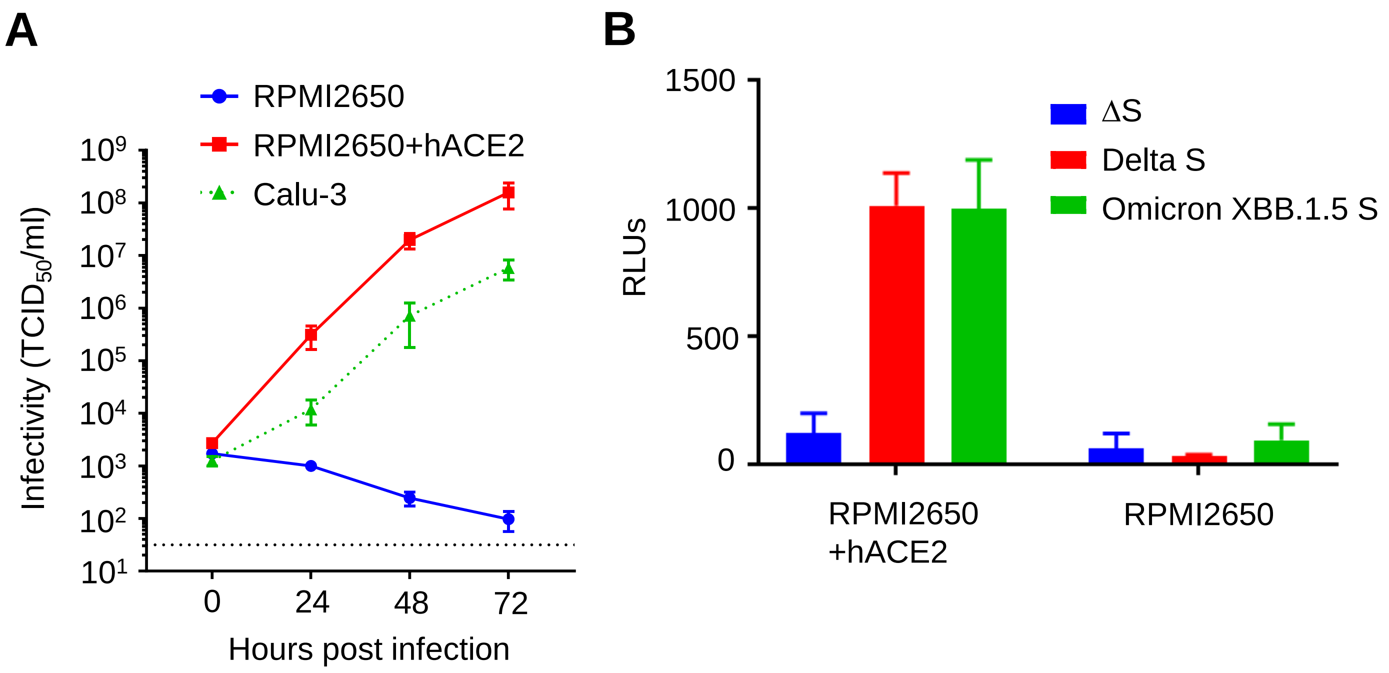

Fig S1 Characterization of established RPMI2650+hACE2 cells. (A) RPMI2650, RPMI2650+hACE2, and Calu-3 cells were infected with SARS-CoV-2 Delta at an MOI of 0.1. After 0, 24, 48 and 72 h of incubation, culture supernatant was collected and subjected to TCID50 assays using VeroE6/TMPRSS2 cells to determine the viral titers (data presented as mean ± SD, n = 3). Each dataset is representative of two independent experiments. (B) RPMI2650 and RPMI2650+hACE2 cells were infected with VSV pseudotyped with spike proteins derived from SARS-CoV-2 Delta or Omicron XBB.1.5. After 1 d of incubation, luciferase activity in infected cells was measured to evaluate the virus infectivity (data presented as mean ± SD, n = 3). Each dataset is representative of two independent experiments.

A

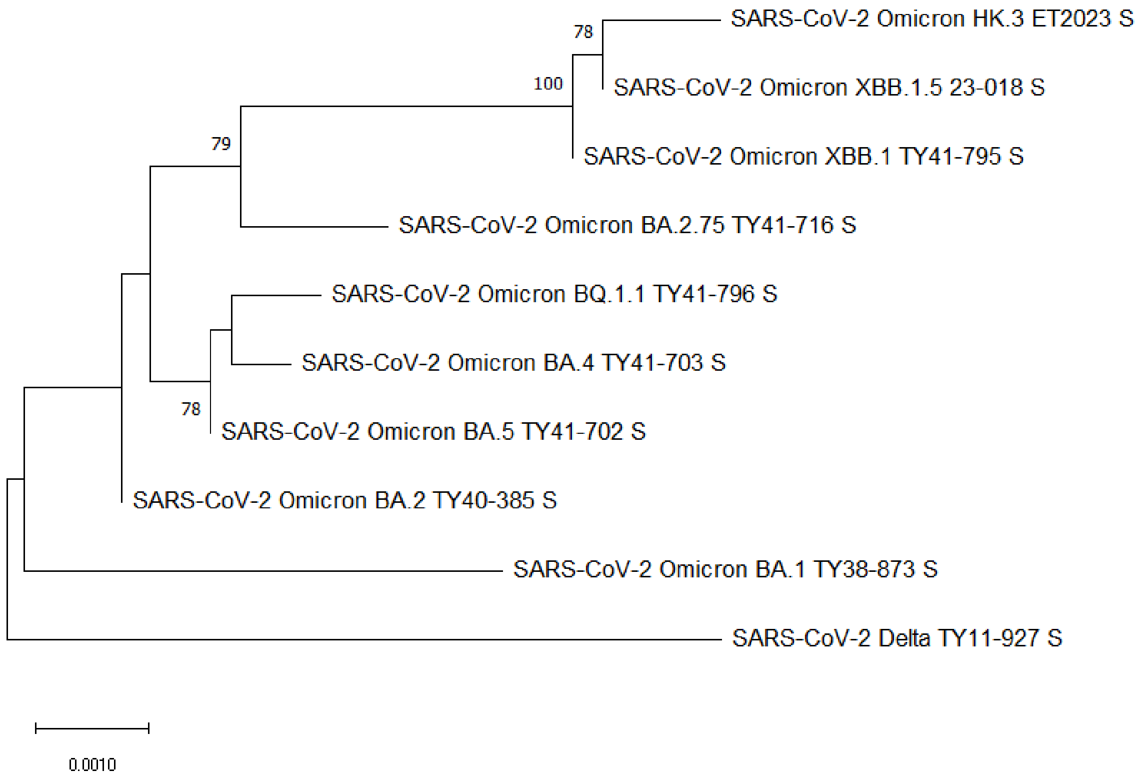

B

Variant/subvariant	Strain name	Isolated date (year-month)
Delta	TY11-927	2021-04
Omicron BA.1	TY38-873	2021-11
Omicron BA.2	TY40-385	2022-01
Omicron BA.5	TY41-702	2022-04
Omicron BA.4	TY41-703	2022-05
Omicron BA.2.75	TY41-716	2022-06
Omicron BQ.1.1	TY41-796	2022-09
Omicron XBB.1	TY41-795	2022-09
Omicron XBB.1.5	23-018	2022-12
Omicron HK.3	ET2023	2023-09

Fig S2 Phylogenetic and chronological information on tested viruses. (A) A maximum-likelihood tree was inferred with S gene sequences of tested SARS-CoV-2 variants. Bootstrap values of ≥70% are shown at the main nodes. A Scale bar indicates nucleotide substitutions per site. (B) Variant/subvariant name, strain name and Isolated date of each tested virus are shown in a table.
